# Forecasting the Walking Assistance Rehabilitation Level of Stroke Patients Using Artificial Intelligence

**DOI:** 10.3390/diagnostics11061096

**Published:** 2021-06-15

**Authors:** Kanghyeon Seo, Bokjin Chung, Hamsa Priya Panchaseelan, Taewoo Kim, Hyejung Park, Byungmo Oh, Minho Chun, Sunjae Won, Donkyu Kim, Jaewon Beom, Doyoung Jeon, Jihoon Yang

**Affiliations:** 1 Machine Learning Research Laboratory, Department of Computer Science and Engineering, Sogang University, 35 Baekbeom-ro, Mapo-gu, Seoul 04107, Korea; seokh@sogang.ac.kr (K.S.); youmeky5@sogang.ac.kr (B.C.); hamsapriya@sogang.ac.kr (H.P.P.); 2 Department of Rehabilitation Medicine, National Traffic Injury Rehabilitation Hospital, 260 Jungang-ro, Yangpyeong-gun, Gyunggi-do 12564, Korea; drcadaver@ntrh.or.kr (T.K.); 20180018@ntrh.or.kr (H.P.); moya1@snu.ac.kr (B.O.); 3 Department of Rehabilitation Medicine, Seoul National University Hospital, Seoul National University College of Medicine, 101 Daehak-ro, Jongno-gu, Seoul 03080, Korea; 4 Asan Medical Center, Department of Rehabilitation Medicine, University of Ulsan College of Medicine, 88 Olympic-ro 43-gil, Songpa-gu, Seoul 05505, Korea; mhchun0@gmail.com; 5 Department of Rehabiliation Medicine, Yeouido St. Mary’s Hospital, College of Medicine, The Catholic University of Korea, 10 63-ro, Yeongdeungpo-gu, Seoul 07345, Korea; gstinfog@catholic.ac.kr; 6 Department of Physical Medicine and Rehabilitation, Chung-Ang University Hospital, Chung-Ang University College of Medicine, 102 Heukseok-ro, Dongjak-gu, Seoul 06973, Korea; donkim21@cau.ac.kr; 7 Department of Rehabilitation Medicine, Seoul National University College of Medicine, Seoul National University Bundang Hospital, 82 Gumi-ro, 173beon-gil, Bundang-gu, Seongnam-si 13620, Gyeonggi-do, Korea; powe5@snubh.org; 8 Department of Mechanical Engineering, Sogang University, 35 Baekbeom-ro, Mapo-gu, Seoul 04107, Korea; dyjeon@sogang.ac.kr

**Keywords:** machine learning, deep learning, classification, stroke rehabilitation, walking assistance device, automated diagnostics, diagnostic reasoning, medical decision making

## Abstract

Cerebrovascular accidents (CVA) cause a range of impairments in coordination, such as a spectrum of walking impairments ranging from mild gait imbalance to complete loss of mobility. Patients with CVA need personalized approaches tailored to their degree of walking impairment for effective rehabilitation. This paper aims to evaluate the validity of using various machine learning (ML) and deep learning (DL) classification models (support vector machine, Decision Tree, Perceptron, Light Gradient Boosting Machine, AutoGluon, SuperTML, and TabNet) for automated classification of walking assistant devices for CVA patients. We reviewed a total of 383 CVA patients’ (1623 observations) prescription data for eight different walking assistant devices from five hospitals. Among the classification models, the advanced tree-based classification models (LightGBM and tree models in AutoGluon) achieved classification results of over 90% accuracy, recall, precision, and F1-score. In particular, AutoGluon not only presented the highest predictive performance (almost 92% in accuracy, recall, precision, and F1-score, and 86.8% in balanced accuracy) but also demonstrated that the classification performances of the tree-based models were higher than that of the other models on its leaderboard. Therefore, we believe that tree-based classification models have potential as practical diagnosis tools for medical rehabilitation.

## 1. Introduction

Cerebrovascular accidents (CVA), i.e., strokes, could lead to walking impairments ranging from mild gait imbalance to complete loss of mobility for patients. Therefore, rehabilitation walking therapy for those patients starts with the proper prescription of walking assistance devices, such as a tilt table, a harness, a (hemi) walker, or a (quarter or single) cane. During the prescription of these devices, the diagnostician’s bias might act as noise that could cause misdiagnosis with unnecessary costs for the patients and the hospitals [1]. Therefore, this paper evaluates machine learning (ML) and deep learning (DL) classification algorithms to confirm whether these models could be supportive tools for diagnosticians by providing suitable predictive performance.

With great advances in ML and DL algorithms (although DL is an area of ML, we separated them for comparison), artificial intelligence (AI) techniques have been applied to various areas of image classification [2,3] to Go [4] and games [5,6]. Especially in the medical domain, numerous studies have also been conducted, including cancer detection with image classification [7], a patient modeling system for clinical demonstration [8], an emergency screening system that differentiates acute cerebral ischemia and stroke mimics [9], a gait monitoring system that predicts stroke disease [10], etc. In the rehabilitation domain, walking assistance robot development [11], AI-based virtual reality rehabilitation [12], and forecasting mortality of stroke patients after complete rehabilitation with tree-based ML models [13] have been studied. Although there exist similar studies [14,15] to ours, the former employed only support vector machines (SVM) [16] for gait classification after extracting features using hidden Markov models [17] and the latter only used lasso regression [18] to prevent overfitting from the small sample size when investigating factors affecting stroke patients’ clinical outcomes and when predicting their discharge scores. Different from these studies, this paper aims to evaluate seven different ML and DL classification models with a dataset of 383 stroke patients to determine which walking assistant devices is the most appropriate for a patient according to their conditions.

## 2. Dataset and Experimental Settings

We conducted an exploratory data analysis to extract the data characteristics. We then preprocessed the data to balance the number of class observations using the undersampling, oversampling, and combined sampling methods. The ML and DL classification models were trained with the original (unpreprocessed) or preprocessed dataset. We obtained a set of performance metrics for each method (i.e., accuracy, precision, recall, F1-score, and balanced accuracy) using five-fold cross validation (5-CV).

### 2.1. Data Description

We collected anonymized data on the walking rehabilitation history of 383 stroke patients (1623 observations) from the following five hospitals: Chung-Ang University Hospital (CAUH), Seoul National University Hospital (SNUH), National Traffic Injury Rehabilitation Hospital (NTIRH), The Catholic University of Korea Yeouido St. Mary’s Hospital (CUYMH), and Asan Medical Center (AMC) from January 2019 to January 2021. Table 1 provides details on the number of patients and observations in the dataset.

**Figure 1:**
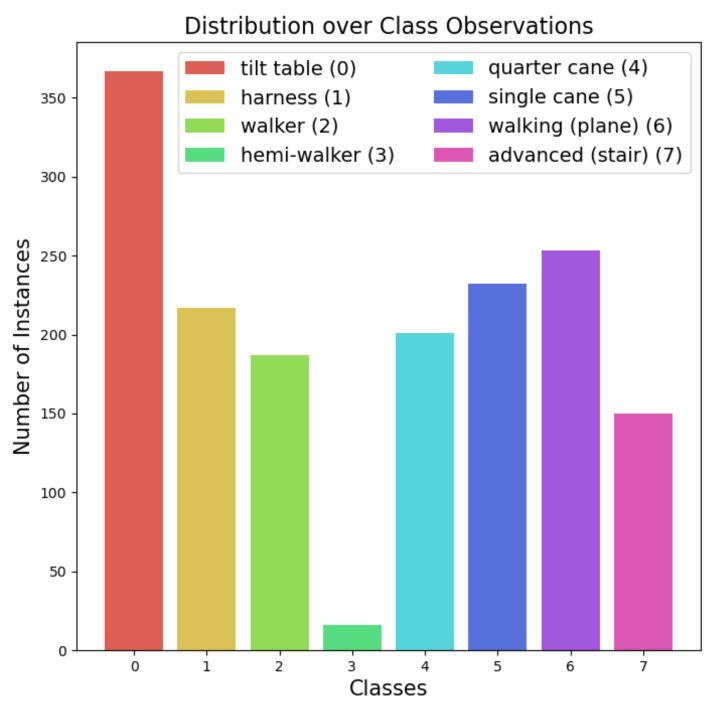
The distribution of the number of observations for eight classes among the collected data. It presents a class imbalance problem, especially for class label 3 (hemi-walker), with only 16 observations.

The features of the data (inputs of the algorithms) were composed of 82 values arranged in six categories: anthropometry, stroke, blood tests, functional assessment, biosignal ward, and disease. We provide the details of the data in Appendix C, including patient characteristics, category distributions, and more specific features in the seven categories. The labels (outputs of the models) were composed of eight classes to differentiate between types of walking assistant devices: tilt table (0), harness (1), walker (2), hemi-walker (3), quarter cane (4), single cane (5), walking (plane) (6), and advanced (stair) (7). Figure 1 displays the distribution of the number of observations for each class.

### 2.2. Data Preprocessing: Undersampling, Oversampling, and Combined Sampling Methods

We adapted three representative sampling methods: SMOTE (over) [19], TomekLinks (under) [20], and SMOTETomek (combined) [21]. We used the imbalanced-learn [22] (Ver. 0.6.2) Python library package, which is compatible with the scikit-learn ML software [23,24]. We provide the backgrounds of the sampling methods in Appendix A.1.

### 2.3. ML and DL Algorithm Settings

For ML, we employed four widely used classification algorithms: SVM [16], Perceptron (PT) [25], Decision Tree (DT) [26], and Light Gradient Boosting Machine (LightGBM) [27]. We also utilized one of the most recently developed automated ML (AutoML) [28] algorithms, the AutoGluon [29] Python library package, to find the best predictive ML classification models with our dataset. For DL, we employed two DL classification models proposed for tabular-formed dataset: SuperTML [30] and TabNet [31]. We also provide their backgrounds in Appendix A.2.

- SVM, PT, and DT settings: we utilized the scikit-learn (Ver. 0.23) [23,24] Python ML library package, and we adapted the radial basis kernel function [32] in SVM and the Gini impurity for a node split criteria in DT. We did not set the regularization term in PT.
- LightGBM settings: in the LightGBM package (Ver. 2.3.1) provided as Python API via scikit-learn [23,24], we empirically decided to use a traditional gradient boosting decision tree as a boosting type without limitations for the number of leaf nodes and depth. We also found that the best performing learning rate was 0.1.
- AutoGluon settings: among the various AutoML Python library packages, we employed the latest and best performing one: AutoGluon (Ver. 0.0.15) [29]. We empirically adjusted the “time_limit” parameter for the whole model from 60 to 120 s and found that the performance did not improve over 120 s. The evaluation metric for each model in the ensemble was set to “accuracy”. We also set the “presets” parameter to be “best_quality” to improve the ensemble models’ predictive performance based on stacking and bagging in the granted training time.
- SuperTML settings: as this model transforms tabular data into images, its performance depends on convolutional structures. Therefore, we experimentally found that ResNet [2] with 152 convolutional layers performed the best.
- TabNet settings: although TabNet [31] is composed of an encoder and a decoder for self-supervised learning [33], we employed only its encoder network for supervised learning. To improve its predictive performance, we modified it into a six-step operation, where we omitted “shared across decision steps” at steps 1–3 under the feature transformer process. We also changed the shared across decision steps to unshared across decision steps in steps 4–6.

### 2.4. Performance Measurement Settings

**Figure 2:**
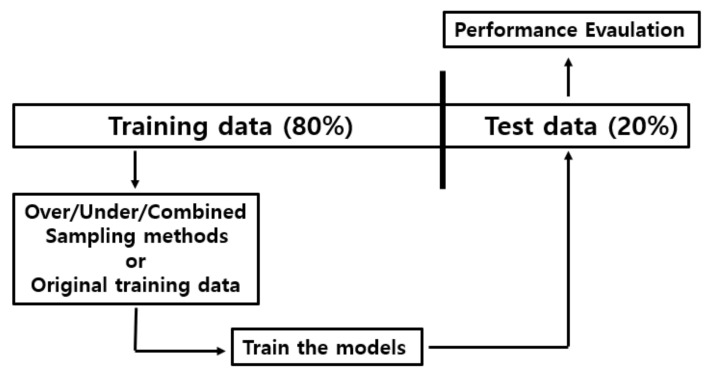
Our evaluation process for the performance of ML and DL algorithms (each step of 5-CV). The collected data were split into 80% for training and 20% for testing. The sampling methods were either applied only to the training data to balance the distribution of class labels or not, after which the models were fitted to the preprocessed data. We then tested them using the test data to evaluate predictive performance.

We measured the classification model’s predictive performance in terms of accuracy, precision, recall, F1-score, and balanced accuracy. As most of these measurements are designed for binary classification problems, we transformed them for multi-class classification using the weighted average conditions in the scikit-learn Python library package [23,24]. We describe the formulations of these measurements in Appendix B. We computed the metrics by averaging the results of 5-CV for fair comparison. In each step of 5-CV, we split all of the data into an 8:2 ratio, where 80% was used for training and 20% was used for testing (validation). For experiments with balanced data, we applied the three sampling methods to the training data, after which the data were used to train the ML or DL models (the models were also trained with the unpreprocessed original data). Finally, the trained models were tested with the test data. Figure 2 summarizes each step of the 5-CV process.

## 3. Results and Discussion

Here, we report and discuss the classification results of the ML and DL models that we employed. We summarize the results in Table 2 via the various classification measurements: accuracy, precision, recall, F1-score, and balanced accuracy.

### 3.1. Classification Results of ML and DL Models

Table 2 presents each model’s classification results according to the data preprocessing methods: original (without sampling methods), SMOTE, TomekLinks, and SMOTETomek. The entries in the table are means and standard deviations, which are denoted in the form mean ± standard deviation. The best accuracy, recall, precision, F1-score, and balanced accuracy among the seven algorithms in each sampling method including the original are highlighted in bold typeface.

In general, the three types of data preprocessing (sampling) methods did not have a positive influence on most classification results except for SVM and SuperTML. Only SVM exhibited dramatic improvements using these methods; for example, an approximately 11% increment was achieved in balanced accuracy by SMOTE and SMOTETomek, whereas only 0.6% was achieved by TomekLinks. On the other hand, SuperTML benefited from SMOTE and SMOTETomek, with only about 0.2% to 1% increments for all results. TomekLinks, however, yielded a reduction in all classification results ranging from 0.2% to 0.7%.

Although most models suffered from a small decline in classification results due to the sampling methods, AutoGluon achieved a more stable predictive performance, where the standard deviations for the averaged 5-CV metrics decreased from 0.3 to 0.2 in accuracy, recall, precision, and F1-score. It seems that, as AutoGluon is an ensemble learning method, some of the newly generated data might positively affect various algorithms within it.

Among the ML and DL classification models, LightGBM and AutoGluon demonstrated the highest classification results (over 90% accuracy, recall, precision, and F1-score). They also presented the highest balanced accuracy: 85% to 86.8%. Note that they all belong to ML classification algorithms and not to DL models. Subsequently, the DL classification models SuperTML and TabNet generated very similar results, with 88.4% to 90.6% accuracy, recall, precision, and F1-score; in contrast, they achieved 82.4% to 85.3% in balanced accuracy. Despite their similar predictive performances, SuperTML required about 70 min of training time whereas TabNet required only about 15 min, which is considered more efficient learning than SuperTML. Finally, it is also notable that the performance results of DT did not reveal much difference from the results of the two DL models, ranging from about 3.4% to 5%. These observations of the results indicate that tree-based ML algorithms are more suitable for our dataset.

### 3.2. Which Model Performed Best?

First, AutoGluon almost always produced the best performance regardless of class distribution (except for balanced accuracy and precision with SMOTETomek sampling). As shown in Table 2, DT, LightGBM, and AutoGluon demonstrated reasonable classification results compared to the other models. In addition, a leaderboard for AutoGluon (Table 3) indicated that the best ranked models are composed of CatBoost boosted trees (CBT) [34], Random Forests (RF) [35], LightGBM, and extremely randomized trees (ERT) [36], which are all tree-based ML algorithms. On the other hand, the DL-based models’ performances were worse than that of LightGBM and AutoGluon. Additionally, they needed longer computational times for 5-CV than the ML models (LightGBM required only 0.09 min and AutoGluon required only 12 min, whereas 15 min were needed for TabNet and 70 min were needed for SuperTML).

The leaderboard of AutoGluon describes the ranking of performance by each classification model based on *Score_test* measured as the log-loss of each model. Notably, the tree-based algorithms in AutoGluon (CBT, LightGBM, RF, and ERT) with different node-splitting criteria (where *Gini, Entr, XT,* and *custom* denote Gini impurity, information gain, extremely randomized, and customized function, respectively) demonstrated the highest classification results, where the score_test values were −0.196,−0.2,−0.223, and −0.228 for CBT, LightGBM, RF, and ERT, respectively. Additionally, the results of DT shown in Table 2 present better classification results than those of other algorithms (SVM and PT). In addition, considering the time spent on the procedure of 5-CV (DT, LightGBM, and AutoGluon took 0.07, 0.09, and 12 min, respectively, whereas 15 min and 70 min were needed for TabNet and SuperTML, respectively), we found that the tree-based classification models are more efficient for learning from our dataset compared to the two DL models, though the performance of DT was 3.4% to 5% lower than that of the DL models.

Additionally, the leaderboard (Table 3) also contains predictive performance of non-tree-based models: K-nearest neighbors (KNN) and neural network classifier (NNC). The Score_test of them exhibited significantly worse (i.e., bigger log-loss) performance relative to CBT (at least a 0.107 difference for NNC and a 0.862 difference for KNN). We further discuss why these tree-based classification models demonstrated better predictive performance than the other models.

**Figure 3:**
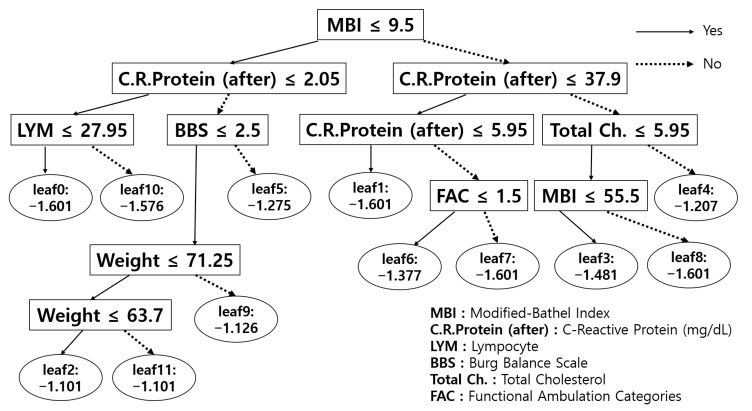
A single LightGBM tree, where each node denotes each feature (square frames) in the dataset and leaf nodes (circular frames) represent the results of classification. For more details on features, refer to Appendix C.

Figure 3 describes a single sample tree from the entire set of trees generated by LightGBM. The square nodes denote features in the dataset, whereas the circular nodes are leaf nodes with raw values before the sigmoid function is applied. The output probability after the sigmoid function indicates that the input observation could belong to some class with the probability value. Generally, most tree-based algorithms define their level of nodes (features) according to various metrics to reduce uncertainty on decision boundaries. In other words, the deeper the level of nodes, the more specific the decision. Once the tree is generated by the training data, the test (unseen) data are classified according to the structures of the trees. We believe that this procedure is very similar to the practical diagnostic reasoning [37] process because the medical diagnostic process is also based on pruning (narrowing) an initial set of hypotheses by gathering more information to lower uncertainties for verification [38,39,40]. Analogous to this, the tree-based models also try to narrow the set of hypotheses by computing and comparing uncertainty-related metrics with each feature to learn the optimal decision boundary. Therefore, due to this similarity, it appears that these tree-based models have an advantage of predictive performance compared to other models.

## 4. Conclusions

In this work, we evaluated the classification performance of ML and DL models for forecasting stroke patients’ walking assistance levels using a dataset gathered from different hospitals. We found that the tree-based ML algorithms yielded the most suitable classification results, and we discussed the similarities between the procedures for tree-based models and actual practical diagnostics. We believe that the similarity is based on the fact that both consist of steps for reducing uncertainty. Based on this similarity, we conclude that tree-based ML classification models are appropriate and competent for medical decision making, including efficient rehabilitation. We expect that tree-based ML or DL models will be applied extensively to other medical domains for alleviating clinicians’ biases during decision making [1] and for developing digital health care platforms, such as *Babylon check* [41].

## Figures and Tables

**Table 1:** Total number of patients and observations for the five hospitals.

	CAUH ${}^{a}$	SNUH ${}^{b}$	NTIRH ${}^{c}$	CUYMH ${}^{d}$	AMC ^e^
The number of patients	29	7	132	173	42
The number of observations	85	34	691	571	242

*^a^* Chung-Ang University Hospital, *^b^* Seoul National University Hospital, *^c^* National Traffic Injury Rehabilitation Hospital, *^d^* The Catholic University of Korea Yeouido St. Mary’s Hospital, and e Asan Medical Center.

**Table 2:** Performance metrics (accuracy, recall, precision, F1-score, and balanced accuracy) of the ML and DL models according to sampling method. We measured recall, precision, and F1-score as weighted averages. The bold typeface stands for the highest metrics in each measurement.

Original Data					
**ML/DL Models**	**Accuracy (%)**	**Recall (%)**	**Precision (%)**	**F1-Score (%)**	**Balanced Accuracy (%)**
SVM	52.1 ± 1.5	52.1 ± 1.5	53.8 ± 2.5	50.4 ± 1.5	41.8 ± 1.5
DecisionTree	86.0 ± 1.0	86.0 ± 1.0	86.4 ± 1.1	86.0 ± 1.1	79.0 ± 1.9
Perceptron	39.1 ± 3.2	39.1 ± 3.2	64.3 ± 2.8	34.7 ± 3.7	32.3 ± 2.6
LightGBM	91.2 ± 0.5	91.2 ± 0.5	91.5 ± 0.5	91.1 ± 0.5	85.8 ± 1.4
AutoGluon	**91.7 ± 0.3**	**91.7 ± 0.3**	**92.0 ± 0.3**	**91.7 ± 0.3**	**86.8 ± 1.3**
SuperTML	89.3 ± 0.8	89.3 ± 0.8	89.8 ± 0.8	89.2 ± 0.9	83.1 ± 2.4
TabNet	89.5 ± 0.6	89.5 ± 0.6	89.8 ± 0.6	89.4 ± 0.6	84.0 ± 1.4
**SMOTE (Over Sampling)**					
**ML/DL Models**	**Accuracy (%)**	**Recall (%)**	**Precision (%)**	**F1-Score (%)**	**Balanced Accuracy (%)**
SVM	57.7 ± 1.6	57.7 ± 1.6	63.9 ± 2.1	59.7 ± 1.8	52.5 ± 3.1
DecisionTree	86.1 ± 0.7	86.1 ± 0.7	86.6 ± 0.7	86.1 ± 0.7	80.7 ± 2.5
Perceptron	38.2 ± 3.6	38.2 ± 3.6	62.7 ± 2.9	35.1 ± 3.6	32.9 ± 2.9
LightGBM	90.8 ± 0.7	90.8 ± 0.7	91.2 ± 0.6	90.8 ± 0.7	86.1 ± 1.2
AutoGluon	**91.0 ± 0.2**	**91.0 ± 0.2**	**91.3 ± 0.2**	**90.9 ± 0.2**	**86.6 ± 1.2**
SuperTML	90.3 ± 0.9	90.3 ± 0.9	90.6 ± 0.9	90.2 ± 0.9	84.1 ± 1.4
TabNet	89.5 ± 0.5	89.5 ± 0.5	90.0 ± 0.5	89.5 ± 0.5	84.9 ± 1.6
**TomekLinks (Under Sampling)**					
**ML/DL Models**	**Accuracy (%)**	**Recall (%)**	**Precision (%)**	**F1-Score (%)**	**Balanced Accuracy (%)**
SVM	53.1 ± 1.6	53.1 ± 1.6	55.2 ± 1.6	51.5 ± 1.8	42.4 ± 1.4
DecisionTree	84.9 ± 0.8	84.9 ± 0.8	85.5 ± 0.8	84.9 ± 0.8	78.6 ± 2.3
Perceptron	35.6 ± 5.7	35.6 ± 5.7	66.5 ± 4.3	32.2 ± 4.3	31.0 ± 3.3
LightGBM	90.0 ± 0.6	90.0 ± 0.6	90.4 ± 0.6	90.0 ± 0.6	85.0 ± 2.5
AutoGluon	**90.2 ± 0.2**	**90.2 ± 0.2**	**90.7 ± 0.1**	**90.2 ± 0.2**	**85.9 ± 1.6**
SuperTML	89.0 ± 0.8	89.0 ± 0.8	89.6 ± 0.8	88.9 ± 0.8	82.4 ± 1.4
TabNet	88.4 ± 0.9	88.4 ± 0.9	88.8 ± 0.8	88.4 ± 0.8	83.0 ± 1.6
**SMOTETomek (Combined Sampling)**					
**ML/DL Models**	**Accuracy (%)**	**Recall (%)**	**Precision (%)**	**F1-Score (%)**	**Balanced Accuracy (%)**
SVM	57.5 ± 1.5	57.5 ± 1.5	63.7 ± 1.5	59.4 ± 1.6	52.5 ± 2.7
DecisionTree	85.7 ± 0.9	85.7 ± 0.9	86.2 ± 1.0	85.8 ± 0.9	80.3 ± 2.4
Perceptron	39.9 ± 3.9	39.9 ± 3.9	62.5 ± 2.4	36.0 ± 4.6	34.3 ± 3.8
LightGBM	**90.4 ± 0.7**	**90.4 ± 0.7**	**90.8 ± 0.6**	**90.4 ± 0.6**	**85.8 ± 1.6**
AutoGluon	**90.4 ± 0.2**	**90.4 ± 0.2**	90.7 ± 0.2	**90.4 ± 0.2**	85.6 ± 1.4
SuperTML	89.8 ± 1.4	89.8 ± 0.9	90.4 ± 0.9	89.8 ± 0.9	83.3 ± 1.7
TabNet	89.2 ± 0.8	89.2 ± 0.8	89.6 ± 0.9	89.2 ± 0.8	85.3 ± 2.7

**Table 3:** Leaderboard for AutoGluon listing the best performing individual classification models from the ensemble model. The attributes Score_test and Score_val are log-loss used to evaluate predictive performance, and the models were sorted according to performance. Note that the closer the value is to zero, the better the model. For details on the other attributes, Stack_level and Fit_order, refer to [29].

Ranking	Model	Score_Test	Score_Val	Stack_Level	Fit_Order
1	CatboostClassifier	−0.196	−0.299	1	22
2	LightGBMClassifierXT	−0.200	−0.293	1	21
3	weighted_ensemble	−0.211	−0.269	2	24
4	LightGBMClassifierCustom	−0.214	−0.345	1	23
5	LightGBMClassifier	−0.217	−0.318	1	20
6	RandomForestClassifierEntr	−0.223	−0.304	1	17
7	ExtraTreesClassifierGini	−0.228	−0.272	1	18
8	ExtraTreesClassifierEntr	−0.231	−0.281	1	19
9	weighted_ensemble	−0.236	−0.319	1	12
10	ExtraTreesClassifierEntr	−0.246	−0.388	0	7
11	ExtraTreesClassifierGini	−0.249	−0.380	0	6
12	CatboostClassifier	−0.254	−0.354	0	10
13	LightGBMClassifierXT	−0.254	−0.347	0	9
14	LightGBMClassifier	−0.270	−0.369	0	8
15	LightGBMClassifierCustom	−0.276	−0.396	0	11
16	RandomForestClassifierGini	−0.278	−0.305	1	16
17	NeuralNetClassifier	−0.303	−0.416	0	1
18	RandomForestClassifierEntr	−0.311	−0.374	0	5
19	NeuralNetClassifier	−0.313	−0.421	1	13
20	RandomForestClassifierGini	−0.318	−0.381	0	4
21	KNeighborsClassifierDist	−1.058	−1.625	1	15
22	KNeighborsClassifierDist	−1.074	−1.757	0	3
23	KNeighborsClassifierUnif	−1.227	−1.767	1	14
24	KNeighborsClassifierUnif	−1.269	−1.901	0	2

## Data Availability

Data sharing is not applicable.

## Appendix A. Background of the Sampling Methods and Classification Models

We provide a brief summary of the conceptional background of the sampling methods and classification algorithms that we evaluated.

### Appendix A.1. Background of the Sampling Methods

- Oversampling (SMOTE): proposed by Chawla et al. [19], the synthetic minority oversampling technique (SMOTE) first chooses a single instance *a* from a minor class at random and arbitrarily selects a single instance *b* that is *k*-nearest to *a*. Then, it draws lines between them, on which a new synthetic instance is generated iteratively via a convex combination of *a* and *b*.
- Undersampling (TomekLinks): the concept of “TomekLinks” is defined via satisfaction of the following conditions, for instance, for *a* and *b* [20]: (1) The two observations are the closest neighbors to each other measured by Euclidean distance. (2) They belong to different class labels (e.g., *a* is in the minor class while *b* is in the major class, and vice versa). Then, the observations in the major class, considered as ambiguous examples, are removed to balance the class distribution.
- Combined sampling (SMOTETomek): Batista et al. [21] empirically demonstrated the effectiveness of the combination of SMOTE [19] and TomekLinks [20]. At first, SMOTE is applied for oversampling. After that, TomekLinks is conducted to remove ambiguous major class observations.

### Appendix A.2. Background of the Classification Methods

- Support vector machines (SVM): SVM for classification [42] aims to find a proper hyperplane that best separates the instances into different classes. In other words, it tries to find a support vector that is orthogonal and maximizes the margin to the hyperplane. SVM uses some kernel tricks to replace the dot product of two vectors with the kernel function.
- Decision Tree (DT): although there are many other tree-based ML algorithms, such as ID3 [43] and C4.5 [44], scikit-learn [23,24] uses the classification and regression trees (CART) [45] algorithm. CART is a binary tree classifier where nodes are split into two child nodes repeatedly with Gini’s impurity index as a splitting criterion. With training data, the decision tree is structured in the direction that reduces Gini’s index.
- Perceptron (PT): PT [46,47] is one of the linear discriminant models for binary classification. The input vector *x* is transformed by a nonlinear transformation to output a feature vector ϕ(x). Then, it is used to construct the following linear model:
  (A1) $$y\left(x\right)=f\left(w^{T}\phi \left(x\right)\right),\;\;\;f\left(a\right)=\left\{\begin{array}{cc} +1 & a\geq 0\\ -1 & a<0 \end{array}\right.$$
  where f(a) is a nonlinear activation function and where target values 1 and −1 correspond to classes 0 and 1, respectively. Then, the stochastic gradient descent algorithm is applied to the perceptron criterion error function to learn the optimal parameter *w*.
- Light Gradient Boosting Machine (LightGBM): LightGBM [27] is a tree-based ML algorithm that utilizes a gradient boosting framework. It is a gradient-based decision tree (GBDT) with two newly proposed techniques to advance the accuracy and efficiency of GBDT (gradient-based one-sided sampling and exclusive feature bundling). With these components, it successfully deals with a large amount of data instances and features efficiently. It grows its nodes in a leaf-wise manner by selecting nodes that decrease loss. This procedure is different from other tree-based ML algorithms, such as GBT [48], GBDT [49], GBM [50], MART [51], and RF [35].
- Automated machine learning (AutoML): AutoML is proposed to automate ML processes such as data preprocessing, algorithm learning, hyperparameter tuning, and evaluation to apply ML to real-world problems. There are two issues regarding AutoML: combined algorithm selection and hyperparameter optimization (CASH) [52], and neural architecture search (NAS) [53]. Between them, we focused on the CASH problem to find the optimal (best-fitted) algorithms for the data collected and drew similarities between the chosen models and the diagnostician’s prescription process in the real world. Although numerous developed AutoML packages exist, we utilized the latest and best performing AutoGluon [29] library package.
- SuperTML: proposed by Sun et al. [30], SuperTML suggested a new way to deal with classification problems using tabular data with deep neural networks by embedding each instance’s features into a two-dimensional image. It then uses a pretrained convolutional neural network (CNN) [54], consisting of residual networks (ResNet) [2], to extract a representation of the images, after which fully connected layers (with two hidden layers) classify the input. It also automatically handles the categorical and missing values without any preprocessing.
- TabNet: similar to tree-based ML algorithms, Arik and Pfister [31] designed a new deep neural network model that performs similarly to the way the tree-based models perform for tabular data (named as TabNet). While the tree-based algorithms efficiently select global features with information gain [26], TabNet also calculates the weights of each instance’s features via step operation. In the step operation, an attentive transformer outputs a mask that is used to take an element-wise product with each batch-sized instance to calculate a sequence of the feature importance. This process belongs to TabNet’s encoder. Although TabNet also has a decoder, it is for unsupervised learning only. That is why we used only the encoder part for supervised learning with six-step operations.

## Appendix B. Formulations of Measurements: Accuracy, Precision, Recall, F1-Score, and Balanced Accuracy

The measurements for evaluating the performance of the classification models are computed as follows:

- Notations: *K*: number of classes, which is 8 in this paper. $C_{i}$: number of observations of class *i*. $TP_{i}$: true positive of class *i*. $TN_{i}$: true negative of class *i*. $FP_{i}$: false positive of class *i*. $FN_{i}$: false negative of class *i*.
- Accuracy: $\frac{\sum_{i=1}^{K}\left(TP_{i}+TN_{i}\right)}{\sum_{i=1}^{k}\left(TP_{i}+TN_{i}+FP_{i}+FN_{i}\right)}$
- Balanced accuracy: $\frac{1}{K}\times \sum_{i=1}^{K}\frac{TP_{i}}{\left(TP_{i}+FN_{i}\right)}$
- Weighted Precision: $\sum_{i=0}^{K}W_{i}\times Precision_{i}$,
  where $\;W_{i}=\frac{C_{i}}{\sum_{j=0}^{K}C_{j}},\;precision_{i}=\frac{TP_{i}}{\sum_{j=0}^{K}\left(TP_{j}+FP_{j}\right)}$
- Weighted recall: $\sum_{i=0}^{K}W_{i}\times Recall_{i}$,
  where $\;W_{i}=\frac{C_{i}}{\sum_{j=0}^{K}C_{j}},\;Recall_{i}=\frac{TP_{i}}{\sum_{j=0}^{K}\left(TP_{j}+FN_{j}\right)}$
- Weighted F1-score: $\sum_{i=0}^{K}W_{i}\times F1_{i}$,
  where $\;W_{i}=\frac{C_{i}}{\sum_{j=0}^{K}C_{j}},\;F1_{i}=2\times \frac{Precision_{i}\times Recall_{i}}{Precision_{i}+Recall_{i}}$

## Appendix C. Details of the Data

We present the collected dataset in a numeric and categorical manner. The numeric variables (in Table A1) are composed of anthropometry, stroke, blood test, functional assessment, and biosignal ward, which are summarized by mean, standard deviation (SD), and range. The categorical ones (in Table A2–Table A6) consist of disease, stroke, and functional assessment, summarized by the number of observations (denoted as ‘#’) and percentages (%).

**Table A1:** Numeric variables and their elements, mean, SD, and value range. The elements are anthropometry, stroke, blood test, functional assessment, and biosignal ward.

Numeric Variables			
**Anthropometry**	**Mean**	**SD**	**Range**
Height (cm)	165.20	8.26	140–190
Weight (kg)	63.19	14.62	0–120
**Stroke**	**Mean**	**SD**	**Range**
National Institute of Health Stroke Scale (NIHSS) initial (h)	0.99	3.99	0–35
NIHSS-tf (h)	0.42	2.47	0–20
**Blood Test**	**Mean**	**SD**	**Range**
Hemoglobin (Hb) (g/dL)	11.87	3.57	0–38
White Blood Cell (WBC) ($10^{6}$/mL)	6.29	3.02	0–40
Lymphocytes (LYM%) (%)	25.93	13.00	0–57
Iymphocyte count ($10^{6}$/mL)	1.42	0.94	0–4.88
Glucose (mg/dL)	95.03	46.40	0–356
C-reactive Protein (before) (mg/L)	4.66	12.49	0–111.38
C-reactive Protein (after) (mg/dL)	40.08	118.82	0–1114
Protein (g/dL)	5.90	2.12	0–8.4
Albumin (g/dL)	3.35	1.14	0–5
Total cholesterol (mg/dL)	76.15	75.04	0–288
**Functional Assessment**	**Mean**	**SD**	**Range**
Modified-bathel index (MBI)	40.90	29.80	0–100
Mini-mental state examination (MMSE)	17.29	15.65	0–330
Modified-ranking scale (mRS)	1.06	1.71	0–5
Burg balance scale (BBS)	20.67	18.04	0–56
NIHSS	0.48	4.15	0–99
**Biosignal Ward (Daily Average)**	**Mean**	**SD**	**Range**
Systolic BP (SBP)	120.22	12.00	89–165
Diastolic BP (DBP)	75.39	10.75	10–120
Heart rate (HR)	77.34	11.06	0–122
Respiratory rate (RR)	19.03	2.03	0–28

**Table A2:** The “disease”-related categorical variables and their elements, the number of observations (#), and percentages (%). The elements are comorbidities and associated impairment.

Categorical Variables					
**Disease**					
** Comorbidities**					
Diabetes Mellitus (DM)	#	%	Chronic Liver Ds.	#	%
Yes	533	33	Yes	28	1.7
No	1090	67	No	1587	97.8
Unknown	0	0	Unknown	8	0.5
Hypertension (HTN)	#	%	Heart Disease	#	%
Yes	1140	70	Yes	248	15.3
No	483	30	No	1367	84.2
Unknown	0	0	Unknown	8	0.5
Chronic Kidney ds. (CKD)	#	%	Hyper Lipidemia	#	%
Yes	45	2.94	Yes	252	15.5
No	1577	97	No	1368	84.3
Unknown	1	0.06	Unknown	3	0.2
Chronic Lung ds.	#	%			
Yes	32	2			
No	1583	97.5			
Unknown	8	0.5			
** Associated Impairment**					
Aphasia	#	%	Neglect	#	%
Yes	493	30	Yes	313	19
No	971	60	No	1229	76
Unknown	159	10	Unknown	81	5
Sensory impairment (light-touch)	#	%	Sensory impairment (pin-prick)	#	%
Intact	547	34	Intact	553	34
Impaired	703	43	Impaired	694	43
Unknown	373	23	Unknown	376	23
Sensory impairment (proprioception)	#	%	Neuropathic pain	#	%
Intact	508	31	Yes	68	4
Impaired	684	42	No	1162	72
Unknown	431	27	Unknown	393	24

**Table A3:** The “stroke”-related categorical variables and their elements, the number of observations (#), and percentages (%). The elements are basic information, lesion location (ischemic), and lesion location (hemorrhagic).

Stroke					
** Basic Information**					
First or Recurred	#	%	Type of stroke	#	%
First-ever Stroke	1492	92	Ischemic	693	42.7
Recurred stroke	131	8	Hemorrhagic	770	47.5
Unknown	0	0	Others	151	9.3
			Unknown	9	0.5
Acute treatment	#	%	First Hospital	#	%
Endovascular Intervention	80	5	Senior General Hospital	5	0.3
Thrombolysis (IA or IV)	111	7	General Hospital	826	50.9
Surgery (burrhole or extraventricular drainage WO craniectomy)	154	9	Hospital	681	42.0
Surgery including craiectomy	324	20	Oriental Medicine Hospital	97	6.0
Medical Treatment	885	55	Others	2	0.1
Others	34	2	Unknown	12	0.7
Unknown	35	2			
Middle cerebral artery (MCA)	#	%	Anterior cerebral artery (ACA)	#	%
Yes	464	28.5	Yes	97	6
No	1149	71	No	1516	93.4
Unknown	10	0.6	Unknown	10	0.6
Posterior Cerebral Artery (PCA)	#	%	Posterior Inferior Cerebellar Artery (PICA)	#	%
Yes	49	3	Yes	146	9
No	1564	96.4	No	1467	90.4
Unknown	10	0.6	Unknown	10	0.6
Anterior Inferior Cerebellar Artery (AICA)	#	%	Corona Radiate	#	%
Yes	55	3.4	Yes	82	5
No	1558	96	No	1531	94.4
Unknown	10	0.6	Unknown	10	0.6
Others	#	%			
Yes	409	25.2			
No	1204	74.2			
Unknown	10	0.6			

**Table A4:** This table belongs to the above “stroke”-related categorical variables.

Lesion Location (Hemorrhagic)					
Frontal	#	%	Temporal	#	%
Yes	128	8	Yes	181	11
No	1495	92	No	1442	89
Unknown	0	0	Unknown	0	0
Parietal	#	%	Occipital	#	%
Yes	97	6	Yes	47	3
No	1526	94	No	1576	97
Unknown	0	0	Unknown	0	0
Basal ganglia	#	%	Brain stem	#	%
Yes	267	16.5	Yes	73	4.5
No	1356	83.5	No	1550	95.5
Unknown	0	0	Unknown	0	0
Intracerebral Hemorrhage (ICH)	#	%	Subarachnoid Hemorrhage (SAH)	#	%
Yes	629	39	Yes	187	11.5
No	994	61	No	1436	88.5
Unknown	0	0	Unknown	0	0
Subdural Hematoma (SDH)	#	%	Intraventricular Hemorrhage (IVH)	#	%
Yes	84	5	Yes	233	14
No	1539	95	No	1390	86
Unknown	0	0	Unknown	0	0
Others	#	%			
Yes	213	13			
No	1410	87			

**Table A5:** The “functional assessment”-related categorical variables and their elements, number of observations (#), and percentages (%). The element are range of motion, modified Ashworth scale, and manual muscle test.

Functional Assessment					
** Range of Motion**					
Right Hip	#	%	Left Hip	#	%
Full	1066	65.7	Full	996	61.4
Limited range in flexion	404	24.9	Limited range in flexion	478	29.5
Limited range in extension	10	0.6	Limited range in extension	8	0.5
Limited range in both directions	124	7.7	Limited range in both directions	122	7.5
Unknown	19	1.1	Unknown	19	1.1
Right Knee	#	%	Left Knee	#	%
Full	1482	91.5	Full	1472	90.7
Limited range in flexion	106	6.5	Limited range in flexion	113	7
Limited range in extension	4	0.2	Limited range in extension	7	0.45
Limited range in both directions	12	0.7	Limited range in both directions	12	0.75
Unknown	19	1.1	Unknown	19	1.1
Right Ankle	#	%	Left Ankle	#	%
Full	1088	67.1	Full	1198	73.8
Limited range in flexion	436	26.8	Limited range in flexion	315	19.4
Limited range in extension	16	1	Limited range in extension	17	1.1
Limited range in both directions	64	4	Limited range in both directions	74	4.6
Unknown	19	1.1	Unknown	19	1.1
** Modified Ashworth Scale**					
Right Elbow Flexor	#	%	Left Elbow Flexor	#	%
0 grade	1389	85.6	0 grade	1285	79.1
1 grade	111	6.8	1 grade	181	11.14
2 grade	105	6.5	2 grade	115	7.1
3 grade	4	0.2	3 grade	27	1.7
4 grade	0	0	4 grade	1	0.06
Unknown	14	0.9	Unknown	14	0.9
Right Knee Flexor	#	%	Left Knee Flexor	#	%
0 grade	1366	84.2	0 grade	1268	78.1
1 grade	177	10.8	1 grade	236	14.5
2 grade	47	2.9	2 grade	82	5.1
3 grade	19	1.2	3 grade	23	1.4
4 grade	0	0	4 grade	0	0
Unknown	14	0.9	Unknown	14	0.9

**Table A6:** This table belongs to the above “functional assessment”-related categorical variables.

Manual Muscle Test					
Right Hip Flexor	#	%	Left Hip Flexor	#	%
Zero	97	6	Zero	88	5.4
Trace	169	10.4	Trace	126	7.8
Poor	302	18.6	Poor	204	12.6
Fair	340	20.9	Fair	303	18.6
Good	510	31.4	Good	524	32.3
Normal	186	11.5	Normal	359	22.1
Unknown	19	1.2	Unknown	19	1.2
Right Knee Extensor	#	%	Left Knee Extensor	#	%
Zero	99	6.1	Zero	94	5.8
Trace	229	14.1	Trace	133	8.2
Poor	231	14.2	Poor	190	11.7
Fair	331	20.4	Fair	294	18.1
Good	522	32.2	Good	533	32.8
Normal	190	11.7	Normal	360	22.2
Unknown	21	1.3	Unknown	19	1.2
Right Dorsi Flexor	#	%	Left Dorsi Flexor	#	%
Zero	110	6.8	Zero	120	7.4
Trace	314	19.3	Trace	300	18.5
Poor	280	17.3	Poor	129	7.9
Fair	200	12.3	Fair	185	11.4
Good	510	31.5	Good	517	31.9
Normal	188	11.6	Normal	353	21.7
Unknown	21	1.2	Unknown	19	1.2
Functional Ambulation Category (FAC)	#	%			
Total Assist	394	24.3			
Maximal Moderate Assist	490	30.2			
Minimal Assist	236	14.5			
Supervision	279	17.2			
Partly Independent	147	9.1			
Fully Independent	56	3.4			
Unknown	21	1.3			
